# The topology of astrocyte networks controls the propagation of intercellular calcium waves

**DOI:** 10.1186/1471-2202-15-S1-P205

**Published:** 2014-07-21

**Authors:** Jules Lallouette, Maurizio De Pittà, Eshel Ben-Jacob, Hugues Berry

**Affiliations:** 1 EPI Beagle, INRIA Rhône-Alpes, Villeurbanne, France; 2 LIRIS, Université de Lyon, UMR5205 CNRS-INSA, Villeurbanne, France; 3 School of Physics and Astronomy, Tel Aviv University, Ramat Aviv, Israel; 4 Center for Theoretical Biological Physics, Rice University, Houston, TX, USA

## 

**Figure1:**
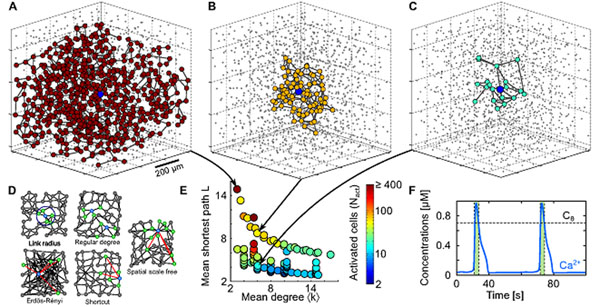
3D representations of activated astrocytes during an ICW with **A**. large extent, **B**. medium extent and **C**. very restricted extent. Gray nodes denote astrocytes that were not activated by the ICW while the blue central node denotes the stimulated astrocyte. **D**. Different topologies of astrocyte networks that were used in this study represented in 2D for readability. **E**. Color-coded ICW extent in the mean degree / mean-shortest path plane. Each point represents 20 realizations of a network. **F**. Stereotypical shape of calcium oscillations in our biophysical model. Green shaded regions denote activation of the astrocyte.

In recent years, astrocytes, one of the main types of glial cells, have been suggested to be active players in neuronal communication. While previously considered to form syncytia with little or no spatial organization, emerging experimental evidence suggests that astrocytes could actually organize into real networks coupled by gap junction channels, with complex topologies that may depend on the brain region. Intercellular calcium waves (ICW) are considered the main pathway for cell-to-cell signaling in these networks. However, it is still not understood why the extent of these ICW depends on the brain region or the experimental protocol considered. To investigate the hypothesis that this variability could actually be linked to the heterogeneous properties of astrocyte networks we studied ICW propagation in biophysically realistic models of three-dimensional astrocyte networks (Figure 1) keeping constant both biophysical properties and spatial distribution of the cells while varying network topology according to different topological schemes (Figure 1D). Numerical simulations revealed that mere changes in network topology could indeed control the extent of ICWs from regenerative ICWs that roughly span the whole network (Figure 1A), to very restricted ones which activate only few tens of astrocytes (Figure 1C). Remarkably, ICW propagation was favored by sparse connectivity (i.e low mean degree) and restriction of cell connections to short distances (i.e large mean-shortest path). Networks with fewer gap junction couplings and stronger distance restrictions on couplings (Figure 1E, *top left quadrant*) supported much larger ICWs than either strongly coupled networks or networks comprising long distance couplings (Figure 1E, *bottom right quadrant*). Our results provide experimentally testable hypotheses to explain several experimental observations and theoretical support to the hypothesis of a functional role for the gap junction couplings in astrocyte networks. In particular, dynamic control of the topology of gap-junction couplings by neuronal activity suggests a novel type of neuron-glia communication.

